# Mechanical Properties of Ti-Nb-Cu Alloys for Dental Machining Applications

**DOI:** 10.3390/jfb13040263

**Published:** 2022-11-22

**Authors:** Masatoshi Takahashi, Kotaro Sato, Genichi Togawa, Yukyo Takada

**Affiliations:** Division of Dental Biomaterials, Tohoku University Graduate School of Dentistry, 4-1 Seiryo-machi, Aoba-ku, Sendai 980-8575, Japan

**Keywords:** titanium alloy, alloy phase, mechanical property, dental alloy, tensile strength, hardness

## Abstract

Titanium has excellent biocompatibility and good corrosion resistance and is extensively used in dental implants and denture bases. However, pure titanium lacks the strength for use in dental prostheses that require relatively high strength. We developed 15 different types of Ti-Nb-Cu alloys and investigated their alloy phases and mechanical properties, including tensile and yield strength, elongation after fracture, and Vickers hardness. The alloy phases of Ti-8%Nb-2%Cu and Ti-13%Nb-2%Cu were α + β, while those of Ti-5%Nb-5%Cu and Ti-10%Nb-5%Cu were α + Ti_2_Cu. The tensile strength and hardness of these alloys were significantly higher than those of titanium; however, their elongation was less. In particular, the yield strength of these alloys was more than twice that of titanium. These differences in mechanical properties are attributable to solid–solution strengthening and precipitation strengthening. Other compositions with an alloy phase of α + β + Ti_2_Cu or β + Ti_2_Cu had high hardness but not high strength. These results suggest that the Ti-8%Nb-2%Cu, Ti-5%Nb-5%Cu, Ti-13%Nb-2%Cu, and Ti-10%Nb-5%Cu alloys can be applied to dental prostheses, which are subject to very high forces from accessories such as long-span bridges, clasps, implant-retained superstructures, and narrow-diameter implants.

## 1. Introduction

Ti, which has excellent biocompatibility and good corrosion resistance, is extensively used in dental implants and denture bases. In Japan, fully cast and resin-veneered Ti crowns have been covered by the medical insurance system since 2020 and 2022, respectively. Ti is expected to replace precious metal and cobalt–chromium alloys in dental applications. However, pure Ti lacks the strength for use in dental prostheses such as bridges and clasps, which require relatively high strength. These prostheses are usually manufactured with high-strength metallic materials classified as types 4 and 5 according to the ISO 22674 criteria, including hardened type 4 dental casting gold alloys and cobalt–chromium alloys [1,2]. Narrow-diameter implants used in cases of insufficient alveolar bone or minimally invasive treatment also require high strength materials [3,4].

Although Ti alloys containing aluminum (Al), vanadium (V), and niobium (Nb), such as Ti-6Al-4V, Ti-6Al-7Nb, and commercially pure grade 4 Ti containing impurities, are available [5], there are concerns regarding the safety of the alloying elements V and Al [6,7]. In addition, the machinability of these alloys is worse than that of pure Ti [8,9], which is difficult to cut and has poor machinability and grindability [10,11]. Pure Ti also has properties that cannot be avoided in casting, such as its high melting point, high reactivity with investment materials at high temperatures, large casting shrinkage, and difficulty in applying casting pressure due to its small specific gravity. Cutting, grinding, and polishing of materials is essential when fabricating dental prostheses. If Ti could be cut easily, it could be used to manufacture dental prostheses using computer-aided design and manufacturing (CAD/CAM). To improve the mechanical properties and machinability of Ti and expand its application in dentistry, we have been developing Ti alloys not containing Al or V [12,13,14,15,16].

We found that the binary Ti alloys made with Ti-Nb and Ti-copper (Cu) have excellent mechanical properties and machinability. The tensile strength, yield strength, and hardness of Ti-Nb alloys increased with increasing Nb content. The strength of 30mass% Nb (hereafter, “%” stands for “mass%”) was more than twice that of Ti, and its elongation after fracture was about 10% [12]. In a grindability test performed using a carborundum wheel, the grinding amount of the Ti-30% Nb alloy was twice that of pure Ti at grinding speeds of 500 m/min and 750 m/min [12]. Further, the strength, yield strength, and hardness of Ti-Cu alloys increased with an increase in the Cu content. The strength of the 5% Cu alloy was more than twice that of Ti, and its elongation was more than 5% [13]. The grinding amount of the Ti-Cu alloy containing 5% Cu was significantly higher than that of Ti at grinding speeds of more than 750 m/min, and it increased significantly at higher speeds [14]. The grinding amount of this alloy at a speed of 1500 m/min was 2.9 times that of Ti. The yield strength and elongation of the 30% Nb and 5% Cu alloys were comparable to those of the hardened type 4 dental casting alloy and cobalt–chromium alloys. They may therefore be applicable in denture bases and clasps. They are also the candidate Ti alloys for CAD/CAM owing to their excellent grindability. In addition, Ti-Cu alloys have good properties such as corrosion resistance, biocompatibility, and antibacterial properties [17,18,19,20,21,22,23].

To further increase the strength and grindability of Ti alloys, we used the elements of the binary alloys to develop ternary alloys. We focused on the three compositions of Ti-6%Nb-4%Cu, Ti-18%Nb-2%Cu, and Ti-24%Nb-1%Cu, which are located between the Ti-30% Nb and Ti-5% Cu alloys on the Ti-Nb-Cu ternary composition diagram. The strength of these Ti-Nb-Cu alloys was higher than that of the base Ti-30% Nb and Ti-5% Cu alloys [24]. In addition, their grindability was superior to that of Ti [25]. Through this series of studies, we discovered the potential of Ti-Nb-Cu alloys for use with CAD/CAM. Ti-Nb-Cu alloys with antibacterial and shape-memory properties have also been developed [26,27].

Phase diagrams are essential for alloy development. Although various binary phase diagrams have been presented, ternary phase diagrams are complex, and only a limited number of element combinations exist. Because no ternary Ti-Nb-Cu phase diagram had been developed before, we constructed a Ti-Nb-Ti_2_Cu pseudo-ternary phase diagram to serve as a development map in our recent study [28]. This phase diagram showed that the Ti-Nb-Cu alloys had phases such as α + β, α + Ti_2_Cu, and α + β + Ti_2_Cu. In general, alloy phases are related to their mechanical properties, and compositions with phases α + β, α + Ti_2_Cu, and α + β + Ti_2_Cu are expected to have good mechanical properties. Therefore, in this study, Ti-Nb-Cu alloys with various compositions and phases were developed and their mechanical properties were investigated.

## 2. Materials and Methods

### 2.1. Alloy Design and Preparation

**Figure 1:**
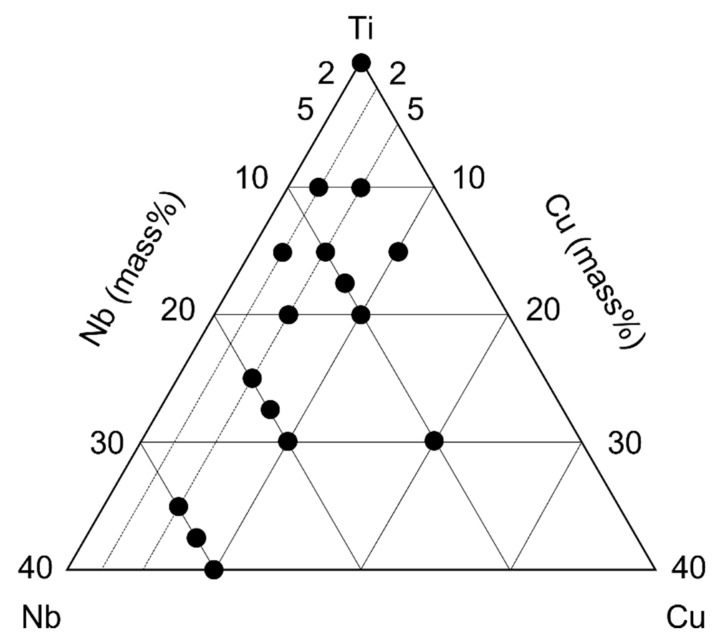
Composition of experimental ternary Ti-Nb-Cu alloys. Black dots represent the compositions designed in this study.

Experimental ternary Ti-Nb-Cu alloys of the following fifteen different compositions were designed; these are the same as those investigated for the construction of the phase diagram in our previous study [28]: Ti-8%Nb-2%Cu (8Nb2Cu), Ti-5%Nb-5%Cu (5Nb5Cu), Ti-13%Nb-2%Cu (13Nb2Cu), Ti-10%Nb-5%Cu (10Nb5Cu), Ti-5%Nb-10%Cu (5Nb10Cu), Ti-10%Nb-7.5%Cu (10Nb7.5Cu), Ti-15%Nb-5%Cu (15Nb5Cu), Ti-10%Nb-10%Cu (10Nb10Cu), Ti-20%Nb-5%Cu (20Nb5Cu), Ti-20%Nb-7.5%Cu (20Nb7.5Cu), Ti-20%Nb-10%Cu (20Nb10Cu), Ti-10%Nb-20%Cu (10Nb20Cu), Ti-30%Nb-5%Cu (30Nb5Cu), Ti-30%Nb-7.5%Cu (30Nb7.5Cu), and Ti-30%Nb-10%Cu (30Nb10Cu) (Figure 1).

The required quantities of Ti (>99.8%, grade S-90, Osaka Titanium Technologies, Amagasaki, Japan), Nb (9.9%, H.C. Starck, Goslar, Germany), and Cu (>99.99%, Research Institute for Electric and Magnetic Materials, Sendai, Japan) were weighed and melted in an argon arc melting furnace to produce a 30 g alloy ingot, as described previously [28]. An ingot of pure Ti was prepared in the same manner.

### 2.2. Preparation of Specimens

The alloy ingots were cast into a mold made of a magnesia investment (Symbion-TC, i-Cast Co., Ltd., Kyoto, Japan) using a titanium casting machine for dentistry (Autocast HC-III, GC, Tokyo, Japan) at 200 °C, followed by bench-cooling. Slabs of dimensions 3.5 mm × 8.5 mm × 30.5 mm were used for the X-ray diffraction (XRD) experiments, hardness tests, and microstructural observations, while specimens with a diameter of 3.0 mm and gauge length of 15 mm were used for the tensile tests. Hardened surface layers of cast slabs were removed by abrading to a depth of 250 µm with 180–800 grit silicon carbide abrasive papers.

### 2.3. Phase Identification

XRD experiments were performed on an X-ray diffractometer (D2 PHASER, Bruker AXS, Tokyo, Japan) over a 2θ range of 30°–80° using Cu Kα radiation (30 kV, 10 mA). The peaks were identified according to the Crystallography Open Database.

### 2.4. Metallography

The cast slabs were cut into specimens of dimensions 8 mm × 8 mm × 3 mm. The specimens were embedded in epoxy resin and only one surface was exposed. The exposed surface was sequentially polished using diamond suspensions with particle sizes of 15, 6, 3, and 1 µm. The mirror-polished surfaces were etched with an etching solution (HF:HNO_3_:H_2_O = 1:4:25) and then observed using an optical microscope (PMG3-614U, Olympus, Tokyo, Japan).

### 2.5. Hardness Tests

The Vickers hardness of the specimens was evaluated using a micro Vickers hardness tester (HM-221, Mitutoyo, Kawasaki, Japan) with a 1.961 N load and 15 s dwell time. Tests (*n* = 9) were performed at three randomly chosen points on each specimen.

### 2.6. Tensile Tests

Tensile testing of each specimen was performed at room temperature at a crosshead speed of 0.5 mm/min on a universal testing machine (AG-IS, Shimadzu, Kyoto, Japan) (*n* = 6). The ultimate tensile strength, yield strength at 0.2% nonproportional extension, and elongation after fracture were determined. The fractured surfaces of the tensile specimens were examined by scanning electron microscopy (SEM; JSM-6060, JEOL, Tokyo, Japan) after testing.

### 2.7. Statistical Analysis

The obtained data were statistically analyzed with SPSS software version 28 using one-way ANOVA and Tukey’s HSD test at a significance level of α = 0.05.

## 3. Results

### 3.1. XRD Findings

Table 1 lists the alloy phases identified by XRD. The peaks in the XRD pattern of the experimental Ti-Nb-Cu alloy in this study matched those of α-Ti, β-Ti, and Ti_2_Cu. The 5Nb5Cu, 5Nb10Cu, and 10Nb5Cu alloys had the α + Ti_2_Cu phase. The 8Nb2Cu, 13Nb2Cu, 15Nb5Cu, and 20Nb5Cu alloys had the α + β phase. The 10Nb7.5Cu, 10Nb10Cu, 20Nb7.5Cu, 20Nb10Cu, and 10Nb20Cu alloys had the α + β + Ti_2_Cu phase. The 30Nb5Cu alloy had a single β phase, and the 30Nb7.5Cu and 30Nb10Cu alloys had a β + Ti_2_Cu phase.

### 3.2. Metallography

**Figure 2:**
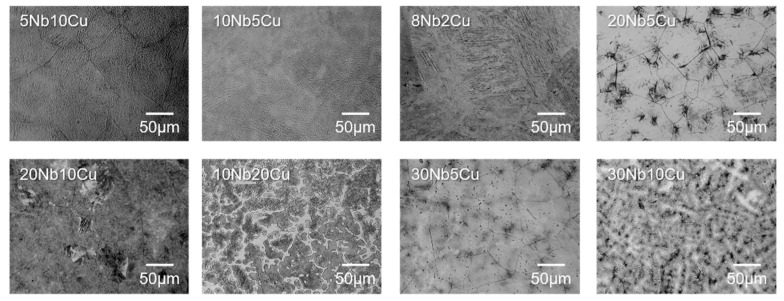
Optical microscopy images of the etched Ti-Nb-Cu alloys.

Among the Ti-Nb-Cu alloys investigated in this study, typical metallographic structures of various alloy phases were selected and are shown in Figure 2. The alloy phases of 5Nb10Cu and 10Nb5Cu were α + Ti_2_Cu. Acicular structures, characteristic of α phase, were observed over the entire surface of the specimen. It was difficult to confirm the precipitation of Ti_2_Cu. The alloy phases of 8Nb2Cu and 20Nb5Cu were α + β. The specimens showed that the acicular α phase precipitated within the equiaxed β grains. The alloy phases of 20Nb10Cu and 10Nb20Cu were α + β + Ti_2_Cu, and precipitated acicular α and Ti_2_Cu were found in the β grains. In addition, the β grains were surrounded by Ti_2_Cu in the 10Nb20Cu specimen. The alloy phase of 30Nb5Cu was a single β; equiaxed β structures were observed over the entire surface of the specimen. The alloy phase of 30Nb10Cu was β + Ti_2_Cu. In this specimen, Ti_2_Cu was precipitated along the β grain boundaries and in the equiaxed β grains.

### 3.3. Vickers Hardness

**Figure 3:**
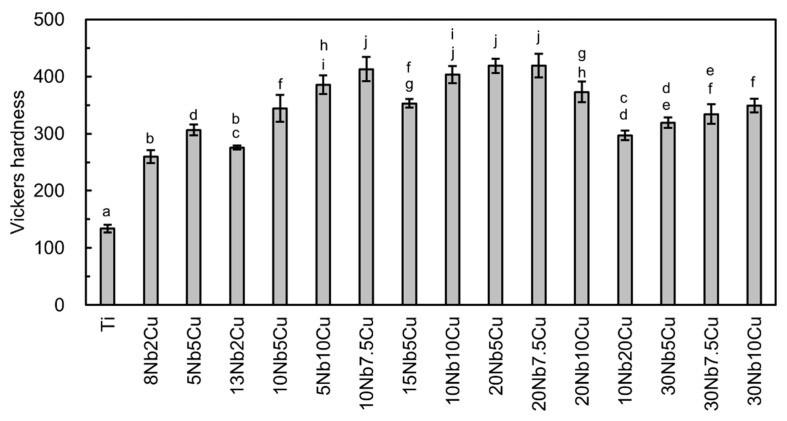
Vickers hardness of the Ti-Nb-Cu alloys. Identical letters indicate a statistically insignificant difference (*p* > 0.05).

Figure 3 presents the Vickers hardness values of the Ti-Nb-Cu alloys. The hardness of all Ti-Nb-Cu alloys was significantly (*p* < 0.01) higher than that of Ti. The hardness values of the 10Nb7.5Cu, 10Nb10Cu, 20Nb5Cu, and 20Nb7.5Cu alloys were particularly high, exceeding 400. When the total amount of the added elements was the same, the alloy hardness increased with increasing Cu content, except for the 20Nb10Cu and 10Nb20Cu alloys.

### 3.4. Tensile Test

**Figure 4:**
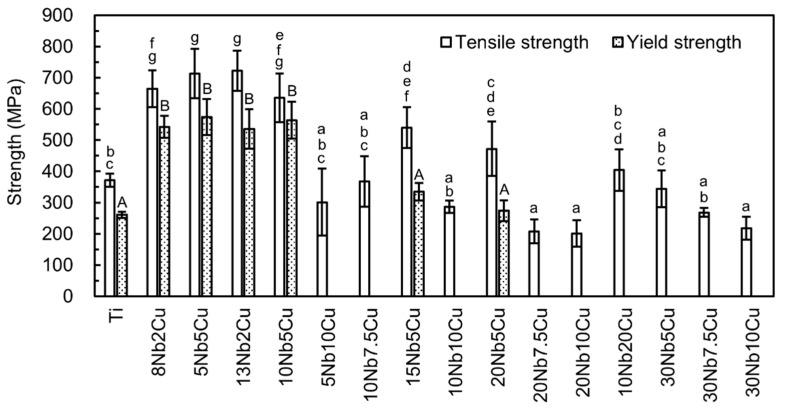
Tensile and yield strengths of the Ti-Nb-Cu alloys. Identical letters indicate a statistically insignificant difference (*p* > 0.05).

Figure 4 shows the tensile and yield strengths obtained. The tensile and yield strengths of the 8Nb2Cu, 5Nb5Cu, 13Nb2Cu, and 10Nb5Cu alloys were significantly greater than those of Ti (*p* < 0.01). No significant difference in the tensile and yield strengths of Ti and Nb-Cu alloys was observed. The tensile strengths of the 5Nb5Cu and 13Nb2Cu alloys were over 700 MPa, more than 1.9 times that of Ti. They had a yield strength of 530 MPa or higher, and the 5Nb5Cu alloy (574 MPa) had the highest yield strength, which was 2.2 times that of Ti. The tensile strengths of the 15Nb5Cu and 20Nb5Cu alloys were significantly greater than that of Ti (*p* < 0.05), but there was no significant difference in the yield strength (*p* > 0.05). The elongations of the six Ti-Nb-Cu alloys were approximately 3%-4%, which was significantly (*p* < 0.01) lower than that of Ti (22%).

The elongation of the Ti-Nb-Cu alloys, other than the above six compositions, was very small (less than 1%). Therefore, their yield strengths could not be determined. The tensile strength of these alloys was less than or equal to that of Ti. The tensile strength of the Ti-Nb-Cu alloys with more than 7.5% Cu content, except for 10Nb20Cu, was lower than that of Ti.

### 3.5. Observation of Fracture Surface after Tensile Test

**Figure 5:**
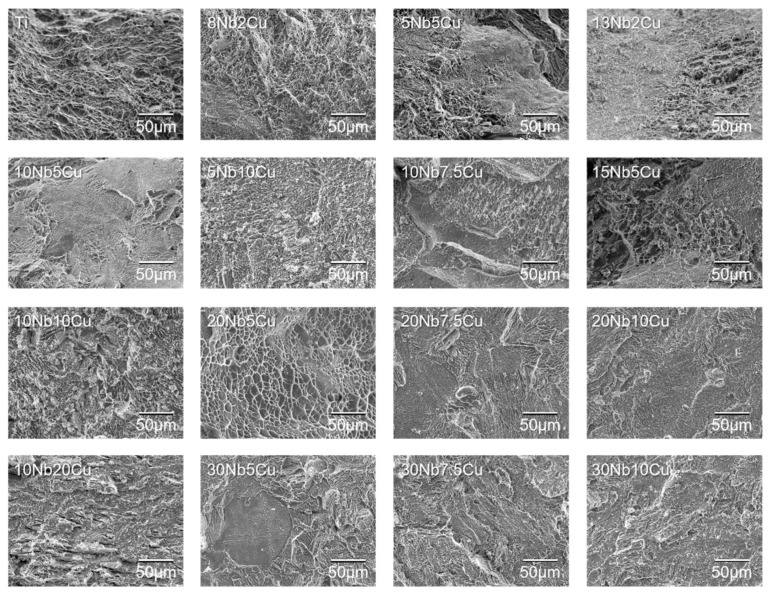
Scanning electron micrographs of the fracture surface of Ti-Nb-Cu alloys after the tensile test.

**Figure 6:**
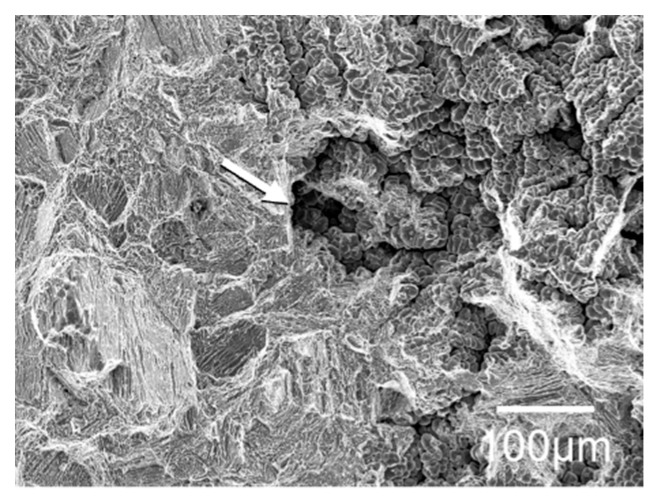
Porosity in the fracture surface of the 5Nb5Cu alloy after the tensile test. The arrow indicates the pores.

Figure 5 shows the SEM image of the fracture surface after the tensile test. Numerous dimples were finely distributed on the fracture surface of ductile alloys with the above six compositions and Ti. The dimples observed on the 20Nb5Cu and 15Nb5Cu alloys were larger than those on the other compositions. A cleavage fracture was observed in the alloy with an elongation of less than 1%. In addition, microshrinkage or many porosities were observed on all specimens. Figure 6 shows an example of porosity.

## 4. Discussion

### 4.1. Alloy Phases and Mechanical Properties

In this study, we investigated the mechanical properties of Ti-Nb-Cu alloys of various compositions developed in our previous study, which was aimed at constructing a Ti-Nb-Ti_2_Cu pseudophase diagram [28]. Therefore, these findings related to the alloy phases and microstructures are consistent with those of the previous study.

**Figure 7:**
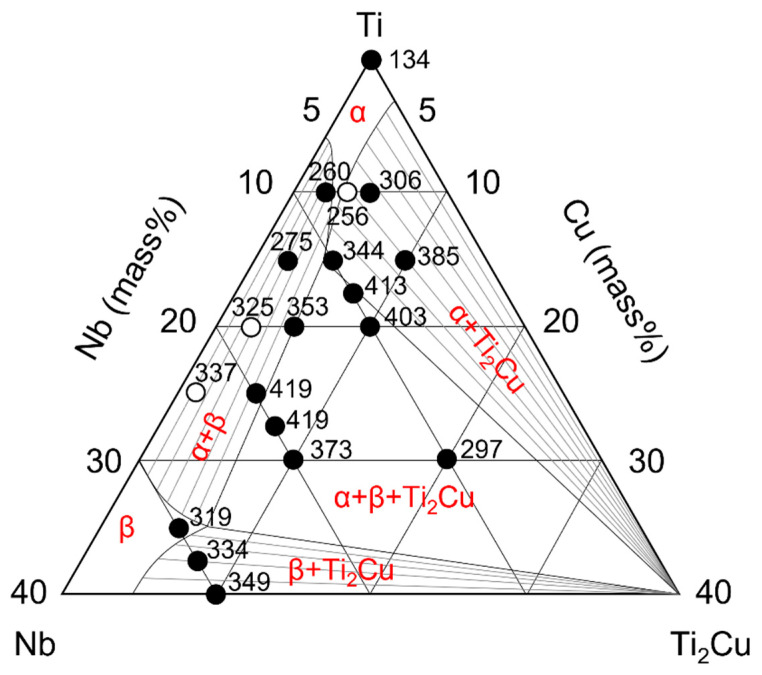
Hardness and alloy phases of the Ti-Nb-Cu alloys. The black dots indicate data obtained in the present study, and the white dots represent data from previous studies.

**Figure 8:**
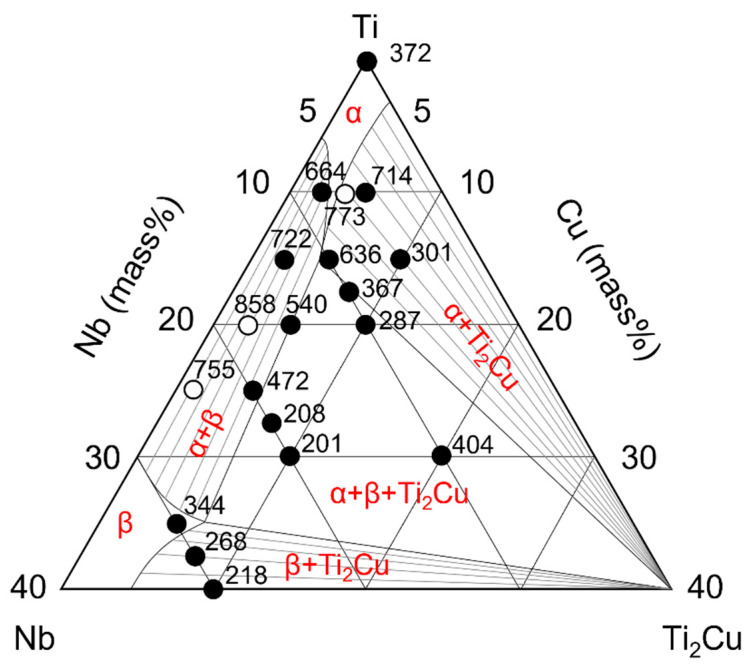
Tensile strength and alloy phases of the Ti-Nb-Cu alloys. The black dots indicate data obtained in the present study, and the white dots represent data from previous studies.

The mechanical properties of metallic materials are closely related to the alloy phases. To further elucidate the relationship between the mechanical properties and alloy phases, the hardness and tensile strength values of the alloys are plotted in the Ti-Nb-Ti_2_Cu pseudophase diagrams in Figure 7 and Figure 8, in which data from previous studies have also been summarized.

Considering the α + Ti_2_Cu alloy phase, the hardness of the 5Nb5Cu alloy was greater than that of the 6Nb4Cu alloy in the α-single phase. This may be attributable to precipitation hardening of Ti_2_Cu in addition to solid solution strengthening of α. The hardness further increased when the amount of solid solution in α-Ti also increased (10Nb5Cu) or the amount of Ti_2_Cu increased (5Nb10C). Its strength was also greater than that of Ti owing to solid solution strengthening and precipitation strengthening. The yield strengths of the 5Nb5Cu and 10Nb5Cu alloys were similar to those of the 6Nb4Cu alloy, but their tensile strength was lower. This may be because precipitation of Ti_2_Cu resulted in a decrease in the elongation. The 5Nb10C alloy, which contains a large amount of Ti_2_Cu, had remarkably less elongation, and its yield strength could not be determined.

In the α + β alloy phase, the hardness increased with increasing the total amount of added elements. Solid solution strengthening was considered to have a strong effect. On comparing the 18Nb2Cu, 15Nb5Cu, 24Nb1Cu, and 20Nb5Cu alloys with the same amount of added elements, the hardness of the two alloys with the most Cu content was found to be greater. Because the phases of these compositions are at the borderline between α + β and α + β + Ti_2_Cu, some Ti_2_Cu undetected by XRD may have precipitated. The strength of the α+ β-type alloy was greater than that of Ti. This two-phase alloy contains acicular α in the residual β phase, resulting in precipitation strengthening. In addition to the solid solution strengthening of the α and β phases, the α + β structure contributes to the high strength. Its strength can be enhanced further by heat treatment [29]. The strength of the 15Nb5Cu and 20Nb5Cu alloys was not significantly high. As described above, this is probably because a small amount of Ti_2_Cu precipitated, and the elongation decreased.

The hardness of the region with the three-phase coexistence of α + β + Ti_2_Cu, except for the 10Nb20Cu alloy, was remarkably high in this study. This may be due to solid solution hardening and precipitation hardening of α and Ti_2_Cu. The hardness of Ti_2_Cu has been reported to be 451 [30]. Because the 10Nb20Cu alloy has the highest Ti_2_Cu content, it was originally predicted to have the greatest hardness. According to the equilibrium phase diagram of the binary Ti-Cu system [31], the peritectic reaction (β Ti) + L → Ti_2_Cu occurs at 1005 °C in the composition range of 17.2% to 43.3% Cu. Most Ti_2_Cu present in the 10Nb20Cu alloy is Ti_2_Cu crystallized by a peritectic reaction during the cooling process of casting. According to previous studies on the microstructure of the 10Nb20Cu alloy, Ti_2_Cu surrounds the β and α grains [28]. Compared with other α + β + Ti_2_Cu alloys, the 10Nb20Cu alloy has abundant Ti_2_Cu, which is mainly crystallized; therefore, the effect of precipitation hardening of Ti_2_Cu on the 10Nb20Cu alloy is small. On the other hand, in relation to the strength of the region with the three-phase coexistence of α + β + Ti_2_Cu, its tensile strength did not improve because extensive precipitation of Ti_2_Cu led to less elongation.

The 30Nb-5Cu alloy was expected to exhibit greater elongation because it is a body-centered cubic crystal of β-Ti, but it did not. Its hardness is greater but its tensile strength is the same as that of Ti. Since a metastable ω phase, which is a brittle phase, was present in the dental cast Ti-30%Nb [12], some ω undetected by XRD may also exist in 30Nb5Cu. Cu acts as a β-phase stabilizer for Ti and shifts the α-β transformation temperature to the low-temperature side, making it easier for the ω-phase to appear. In addition, because the 30Nb5Cu alloy is located at the boundary between the β and β + Ti_2_Cu phases, it is possible that it contained Ti_2_Cu in amounts not detected by XRD. Ti_2_Cu precipitated when the amount of Cu added to the 30Nb5Cu alloy increased, and the alloy phase changed to β + Ti_2_Cu. Precipitation of Ti_2_Cu in the β matrix increased its hardness, but its strength decreased owing to the decrease in elongation. Other Ti-Nb-based ternary alloys, such as Ti-Nb-tin (Sn), Ti-Nb-molybdenum (Mo), and Ti-Nb-iron (Fe) alloys, have also been developed to enhance the performance of the Ti-Nb alloys of β titanium. Sn increases the strength of Ti-Nb alloys and lowers the elastic modulus of the alloy [32]. Fe increases the strength of Ti-Nb alloys, although the ω phase may precipitate depending on the composition and render the alloy embrittled [33,34]. Mo enhances the superelasticity of Ti-Nb alloys [35]. Thus, the various added elements have different roles and influence the alloy properties differently.

### 4.2. Dental Application of Ti-Nb-Cu Alloys

For dental application, the alloys must demonstrate elongation after fracture. ISO 22674 requires alloys to have an elongation of 2% or more [2]. Among the Ti-Nb-Cu alloys examined in this study, dimples, which are characteristic of ductile fracture, were observed on the fracture surfaces of compositions that satisfied the ISO criterion. The dimple size is affected by the properties of the matrix, second-phase particles, relative strength between the matrix and second-phase particles, and bonding strength of the interface [36]. The dimple size on the fracture surface of the compositions near β (20Nb5Cu and 15Nb5Cu) was larger than that near α (8Nb2Cu and 13Nb2Cu). The fracture surface images of the 20Nb5Cu alloy were very similar to those of the 18Nb2Cu and 24Nb1Cu alloys obtained in a previous study [24]. The fracture surface appeared to follow the main matrix phase. Although the elongation of the experimental alloys in this study was approximately 3–4%, which is by no means large, it is close to the elongation (approximately 3–5%) shown by typical α + β, Ti-6Al-4V, and Ti-6Al-7Nb alloys [37,38]. Because microshrinkage or porosities were observed in all samples, it is possible that the elongation was small owing to their influence. Alternatively, the solid solution of oxygen in the alloy during casting may have reduced the elongation. The melting point of Ti alloys decreases with the addition of Cu but increases with the addition of Nb [31]. A composition with high Nb content may have a higher melting point than Ti, which implies that it takes longer to melt, increasing the risk of oxidation during melting for casting. Based on this, the Ti-Nb-Cu alloy may be unsuitable for casting. Wrought Ti-Nb-Cu alloys should exhibit greater elongation. Machining wrought Ti-Nb-Cu alloys with CAD/CAM milling will result in better-performing prostheses. Although the machinability of the Ti-Nb-Cu alloys designed in this study was not investigated, some recently investigated Ti-Nb-Cu alloys exhibited improved grindability [25]. A common feature of Ti alloys with excellent grindability is the presence of a second phase instead of a single α phase [39]. The ductile Ti-Nb-Cu alloys in this study were α + β or α + Ti_2_Cu and were expected to have good machinability.

Considering the ISO criteria for metal dental materials, the yield strength and elongation of the 8Nb2Cu, 5Nb5Cu, 13Nb2Cu, and 10Nb5Cu alloys match types 4 and 5 and can be used for large prostheses and implant superstructures. In addition, the tensile strength of these alloys is equivalent to that of commercially pure grade 4 Ti (550–750 MPa) and American Society for Testing and Materials/American Society of Mechanical Engineers grade 4 (550 MPa or higher) used for narrow-diameter implants [1,40]; therefore, it is likely that these alloys could be used for narrow-diameter implants. The yield strengths of the 15Nb5Cu and 20Nb5Cu alloys satisfied the ISO type 3 criterion, but their elongation was insufficient. The hardness of the 8Nb2Cu and 13Nb2Cu alloys was comparable to that of hardened type 4 dental gold casting alloys (237–264) [1], and that of the 5Nb5Cu and 10Nb5Cu alloys was equal to or slightly lower than that of Ti-6Al-4V alloys (320–341) [1,41]. The hardness of the 15Nb5Cu and 20Nb5Cu alloys was equivalent to that of cobalt–chromium alloys (350–390) [1]. All Ti-Nb-Cu alloys had suitable hardness for dental applications.

Compositions with less than 1% elongation are unsuitable for clinical application. Among the Ti-Nb-Cu alloys described in this study, compositions with a Cu content of 7.5% or more did not exhibit sufficient elongation regardless of the Nb content. No dimples were observed on the fracture surfaces of these compositions after the tensile test, and either cleavage or pseudo-cleavage fractures were observed. Binary Ti-Cu alloys have shown a large drop in elongation at 5% Cu content and in brittleness at 10% Cu [13]. As an alloying element for Ti, Cu can effectively improve its strength and hardness with the addition of some Ag or Au, but the decrease in elongation is remarkable [13]. Therefore, the upper limit for the Cu content in the alloy should be 5%. The 15Nb5Cu and 20Nb5Cu alloys, in which dimples were observed, may exhibit greater elongation. If the elongation of these alloys increased slightly, they would match the ISO type 3 criteria, making them applicable in dentistry.

Copper cytotoxicity may be a concern for dental applications of Ti-Nb-Cu alloys. However, according to Takada et al., although the amount of Cu ions released from Ti_2_Cu was higher than that released from αTi in the NaCl solution, the amount released from Ti_2_Cu was 1/10 times smaller than that from type 4 gold alloys under the same conditions [18]. In addition, many studies aiming at implant applications have been conducted on binary Ti-Cu alloys, and good results have been achieved in many cases [21,22,23]. Therefore, Ti-Nb-Cu alloys should be suitable for dental applications.

## 5. Conclusions

In this study, we developed Ti-Nb-Cu alloys with an aim of improving their machining characteristics for dental applications. The 8Nb2Cu, 5Nb5Cu, 13Nb2Cu, and 10Nb5Cu Ti alloys demonstrated good potential for use in fabricating high-strength dental prostheses. We plan to investigate the machinability of these alloys in the future.

## Figures and Tables

**Table 1:** Alloy phases in Ti-Nb-Cu alloys identified by X-ray diffractometry.

Composition	Alloy Phase
Ti-8%Nb-2%Cu	α + β
Ti-5%Nb-5%Cu	α + Ti_2_Cu
Ti-13%Nb-2%Cu	α + β
Ti-10%Nb-5%Cu	α + Ti_2_Cu
Ti-5%Nb-10%Cu	α + Ti_2_Cu
Ti-10%Nb-7.5%Cu	α + β + Ti_2_Cu
Ti-15%Nb-5%Cu	α + β
Ti-10%Nb-10%Cu	α + β + Ti_2_Cu
Ti-20%Nb-5%Cu	α + β
Ti-20%Nb-7.5%Cu	α + β + Ti_2_Cu
Ti-20%Nb-10%Cu	α + β + Ti_2_Cu
Ti-10%Nb-20%Cu	α + β + Ti_2_Cu
Ti-30%Nb-5%Cu	β
Ti-30%Nb-7.5%Cu	β + Ti_2_Cu
Ti-30%Nb-10%Cu	β + Ti_2_Cu

## Data Availability

The authors confirm that data supporting the findings of this study are available within the article.
